# Adult Ileo-Ileal Intussusception Caused by Intestinal Lipoma

**Published:** 2014-11

**Authors:** Mohammad Sadegh Fazeli, Alireza Kazemeini, Fezzeh Elyasinia, Reza Parsaei

**Affiliations:** Department of Surgery, Tehran University of Medical Sciences, Tehran, Iran

## Dear Editor,

**Figure 1:**
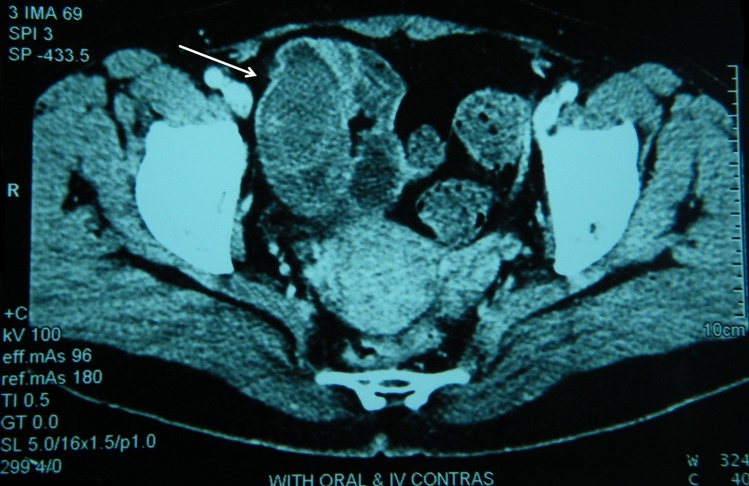
Abdominal CT-Scan shows thickening and invagination of a bowel segment into distal portion (white arrow).

Intussusception is an invagination of a portion of bowel into distal segment. While common in children, it is a rare cause of bowel obstruction in adults and usually is caused by a lead point. Most causes of adult small bowel intussusceptions are benign intestinal lesions like lipoma, leiomyoma, neurofibroma, inflammatory polyps, and meckel diverticulum.^1^ Patients usually present with bowel obstruction or vague abdominal symptoms. Diagnosis is not usually achieved clinically or by laboratory data, but CT imaging is helpful for diagnosis.^2^ Our patient is a 45-year-old woman presented with intermittent abdominal pain. Physical examination was unremarkable and abdominopelvic sonography and upper GI endoscopy were normal. Abdominopelvic CT-Scan performed during an episode of pain showed thickening and invagination of a bowel segment (figure 1). The patient underwent surgery and an Ileo-Ileal intussusception was noticed approximately 100 cm to ileocecal valve invagination was reduced. A palpable mass was found in Ileum. Resection and anastomosis of affected bowel were done. The patient postoperative course was uneventful. Histopathological examination reported a sub mucosal lipoma of ileum.
